# The Intelligent Control System and Experiments for an Unmanned Wave Glider

**DOI:** 10.1371/journal.pone.0168792

**Published:** 2016-12-22

**Authors:** Yulei Liao, Leifeng Wang, Yiming Li, Ye Li, Quanquan Jiang

**Affiliations:** Science and Technology on Underwater Vehicle Laboratory, Harbin Engineering University, Harbin, Heilongjiang, China; West Virginia University, UNITED STATES

## Abstract

The control system designing of Unmanned Wave Glider (UWG) is challenging since the control system is weak maneuvering, large time-lag and large disturbance, which is difficult to establish accurate mathematical model. Meanwhile, to complete marine environment monitoring in long time scale and large spatial scale autonomously, UWG asks high requirements of intelligence and reliability. This paper focuses on the “Ocean Rambler” UWG. First, the intelligent control system architecture is designed based on the cerebrum basic function combination zone theory and hierarchic control method. The hardware and software designing of the embedded motion control system are mainly discussed. A motion control system based on rational behavior model of four layers is proposed. Then, combining with the line-of sight method(LOS), a self-adapting PID guidance law is proposed to compensate the steady state error in path following of UWG caused by marine environment disturbance especially current. Based on S-surface control method, an improved S-surface heading controller is proposed to solve the heading control problem of the weak maneuvering carrier under large disturbance. Finally, the simulation experiments were carried out and the UWG completed autonomous path following and marine environment monitoring in sea trials. The simulation experiments and sea trial results prove that the proposed intelligent control system, guidance law, controller have favorable control performance, and the feasibility and reliability of the designed intelligent control system of UWG are verified.

## Introduction

Traditional motion monitoring platforms (research vessel, unmanned surface vehicle, underwater vehicle etc.) take fuel oil or battery as power sources and the endurance is limited by energy capacity. It consumes lots of time, costs a lot and may bring pollution to the environment. However, there are abundant green energy resources in the oceans. In recent years, more and more researchers pay attention to take ocean energy as the power sources of ocean vehicles. There have been many researches on ocean energy powered unmanned vehicles [1], mainly about solar powered underwater vehicles [2], thermal powered underwater vehicles [3], wind powered or solar-powered unmanned surface vehicles [4,5], and wave powered vehicles.

Unmanned Wave Glider(UWG) is a new kind of wave powered unmanned ocean vehicle. UWG has many outstanding advantages like infinite endurance, autonomy, zero discharge, low cost. And UWG can perform a wide range of tasks such as marine environment monitoring, weather forecast, biological investigation, communication relay, distant early-warning in long time scale and large spatial scale autonomously. UWG has broad application foreground in military and civil fields, and is widely applied in marine scientific research and survey [6–13]. In recent ten years, UWG becomes a hotspot in the world.

The intelligent control system is the most important part for UWG. A control system with good control performance and intelligence level is a precondition for UWG in application. The thrust of UWG comes from wave motion and changes randomly with sea condition. It means that the thrust is random and uncontrollable, and the speed is uncontrollable. Only the rudder system is controllable. Compared to traditional unmanned vehicles, the velocity of UWG is low which causes bad steerage. The UWG control problem is a special kind of weak maneuvering control problem. The Liquid Robotics Co. has achieved remote autonomous control of UWG, but there is no technical detail about the control system and control algorithm.

In 2011, Smith et al. [14] discussed the online speed predicting of UWG. Parameters of the speed predicting were obtained by least square method. But the deviation between numerical predicting result and test data was relatively large. In 2013, Ngo et al. [15] established a nonlinear, random and non-parametrical speed predicting model. The parameters of the model were obtained by Gaussian Process Regression method. Contrast test show that the proposed method can predict the actual speed of UWG effectively. In 2014, Ngo et al. [16] predicted the speed of UWG using Gaussian Process Regression method based on the WAVEWATCH III wave model and observational data. Analysis shows that the resultant accuracy of predicted speed depends on the temporal resolution of the predictive model and training data.

In 2011, Kraus and Bingham [17] established a dynamic model in vertical plane under the assumption that the umbilical is always in tension. And then the vertical motion of UWG was predicted using Extended Kalman Filter. In 2012, considering about the special structure of UWG, Kraus [18] established a six degree maneuvering model based on Ship’s maneuverability theory. PID control algorithm was applied in UWG station keeping control and the mathematical simulation without current was carried out. The results show that the UWG’s station keeping radius is less than 40m and there is a dead zone of 10m radius. Obviously, due to the weak maneuvering property, it is difficult for UWG to realize the same control precision as traditional ocean unmanned vehicles. It provides a foundation to analyze the special motion mechanism of UWG, while trial result shows that the error need to be further reduced.

In 2014, Zhang S et al. [19] developed a shore-based monitoring application program for UWG under Visual Studio C#.net development suite. GPS/Beidou dual-mode technology and Google earth COM API were adopted in the application program. The application program can realize GPS positioning, satellite data transmission, vector map based track displaying, trajectory presetting, environment monitoring data storage and classification, visual checking and querying of working status, etc. The trial results show that the shore-based monitoring system basically completed the instruction sending and trajectory tracking tasks.

In the same year, Shi J et al. [20] mainly discussed the motion control problem of UWG. A LPC2478-based embedded control system was designed. Based on Gaussian geodesic algorithm and PID control algorithm, the navigation strategy was constituted and achieved visual tracking and virtual anchor of UWG. Meanwhile, to ensure the accuracy of navigation information, two groups of sensors (wave height meter and flow meter) were added as auxiliary navigation. A complete navigation system was constructed and sea trials were conducted.

In 2015, Lu X [21] focused on the “Ocean Rambler” UWG. Considering about the nonlinear, time-variable, uncertain properties, a fuzzy adaptive PID algorithm was put forward combining PID control with fuzzy control. Simulation experiments show that the fuzzy PID controller has the advantages of small overshoot and short adjustment time compared with PID controller. And the station keeping simulation experiment was carried out.

In this paper, the architecture of the UWG embedded control system is presented on hardware and software. Considering about the disturbance of the marine environment on the motion of the carrier, a self-adapting PID guidance law is proposed. For the characteristics of difficult modeling and large disturbance, we discuss a heading control method based on S-surface controller. Finally, the efficiency and reliability of proposed methods are verified by tank trials.

## Design of the UWG intelligent control system

We will start with a general description of “Ocean Rambler” UWG before discussing the control system in detail.

### “Ocean Rambler” UWG

**Fig 1:**
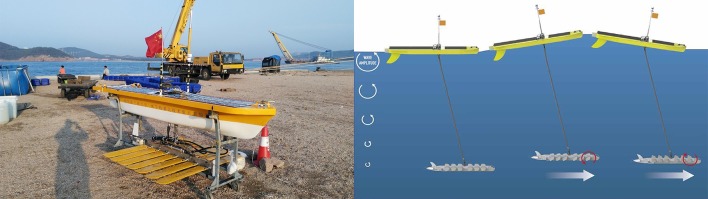
The “Ocean Rambler” UWG and the operating principle.

Since 2012, the Harbin Engineering University Science and Technology on Underwater Vehicle Laboratory has carried out research on UWG firstly in China. Two types of prototypes “Ocean Rambler I” and “Ocean Rambler II” (Fig 1) have been made. Several tank trials and sea trials have been completed.

The UWG consists of a surface float (float), a submerged glider (wave thruster, glider) and a flexible umbilical. Several wings are hinged on the glider. Glider provides thrust for UWG. In wave environment, when the float moves down a wave, the glider falls under its own weight, and the aft of wings rise; conversely, when the float is pulled upward by a wave, the umbilical lifts the glider and the aft of wings fall. The wings fall and rise alternately like a fish that push the glider forward, and the float is pulled forward by umbilical. The operating principle is shown in Fig 1. The UWG mechanically converts the wave energy to the thrust, and other motive powers are unnecessary.

This paper focuses on the “Ocean Rambler” UWG. The UWG is able to sail and work autonomously and separately. Besides, the UWG lays the platform foundation of multiprocessor control like multi-UWGs navigation and teamwork.

### Design of the intelligent control system architecture

#### Analysis of intelligent behavior characteristics

It is challenging to design the control system of UWG. The embedded computer and many sensors should be organized well to make the UWG complete tasks. Large amounts data transfers between the parts inside the control system through data cable or wirelessly. There have been many researches focus on the complex control systems and the communication systems. Some scholars have made outstanding contributions and proposed novel and effective approaches. Cordeschi N et al. [22] discuss the connection between car smartphones and vehicular access networks. The afforded resource management problem is translated into a suitable constrained stochastic network utility maximization problem. The optimal cognitive resource management controller is derived to dynamically allocates the access time-windows at the serving roadside units together with the access rates and traffic flows. Ahmed SH et al. [23] analyze the propagation behavior of interest and data packets in the vehicular ad hoc network environment through extensive simulations. The “CODIE” scheme is applied to control the data flooding/broadcast storm. Simulation results show the CODIE forwards fewer copies of data packets processed while achieving similar interest satisfaction rate compared with the naïve VNDN. Shojafar M et al. [24] address the resource scheduling problem in a grid computing environment. A new approach to resource scheduling in grid computing environments named hierarchical stochastic Petri net (HSPN) is proposed. The proposed approach optimizes grid resource sharing by categorizing resource requests in three layers, where each layer has special functions. The results show the validity of HSPN. Du QH et al. [25] focus on the limited wireless resources in e-health. In order to extract key knowledge from massive access attempts, a hybrid periodic-random massive access scheme for wireless clinical networks employing ultra-narrow band technique is proposed. Simulation results shows that it can effectively enhance the channel utilization efficiency and average packet drop ratio and improve the performance of the network in some extent.

UWG is designed to receive the tasks from shore-side and execute tasks automatically. For example, the UWG may be asked to monitor environment on a planned course or track the hurricane. UWG must be able to understand the tasks, precept the environment, make decision, and finally complete the tasks. The behavior characteristics of UWG are really similar to a human. In this paper, we plan to establish the neuropsychological structural model based on cerebrum basic function combination zone theory. The intelligent robot system can be divided into two parts [26]: physical system and nervous system. The nervous system consists of deliberating region, cognition region, perception region, and each region consists of three cortexes. This model lays the foundation to describe the neuropsychological architecture, cognitive mental mechanism and intelligent behavior process of intelligent robots. Research has shown that the autonomy, responsiveness, initiative, and sociability principles can be taken as the evaluation criterions of the architectural function of intelligent robots. Some typical architectures have been analyzed contrastively including hierarchical degrade, comprehensive, three-layers and social robot architectures, and it shows the several architectures mentioned above lack of supports to initiative and sociability, while the neuropsychological structural model satisfies all the four principles [26].

UWG has the following features which is same as intelligent robots: (1) autonomy—autonomous operation capacity, which means the UWG can decides its behavior by itself; (2) responsiveness—the self-adaptive ability to react instantly to changes of the environment and states of other members; (3) initiative—initiative operation capacity, which means that UWG can take goal-oriented intelligent behaviors driven by its mind without command and intervention from outside; (4) sociability—the cooperation ability should be considered in combined application with UWG, AUV, USV, UAV, etc. which involves cooperation, commitment, coordination, and communication mechanism.

To better describe the features of UWG’s behaviors, we establish the architecture of UWG intelligent control system based on neuropsychological structural model.

#### System composition

**Fig 2:**
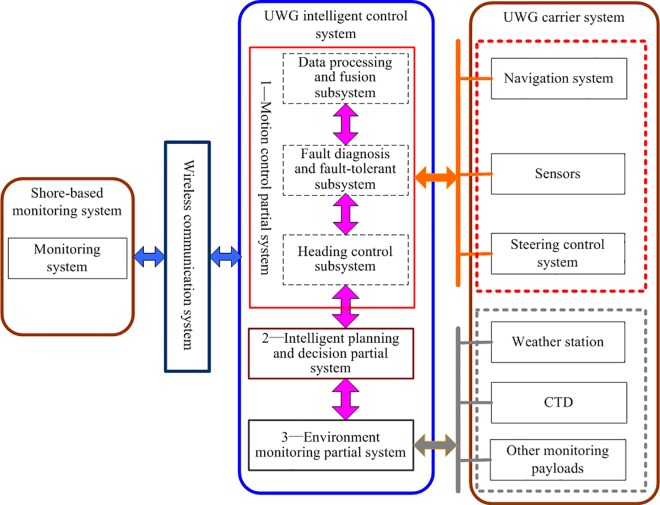
The system composition of UWG.

We divide the UWG into two partial systems: carrier system and nervous system. The carrier system consists of: the carrier of UWG; actuator—the rudder control system; perception system—GPS, magnetic compass, underlying sensors, environment monitoring modules; communication system—radio station, wireless network, satellite communication modules; embedded computer system. By functionality and modularization, the UWG can be divided into four parts: carrier system, intelligent control system, shore-based monitoring system, and wireless data communication system, as shown in Fig 2. The intelligent control system consists of three partial systems: intelligent planning and decision, motion control, and environment monitoring.

#### Neuropsychological structural modeling

**Fig 3:**
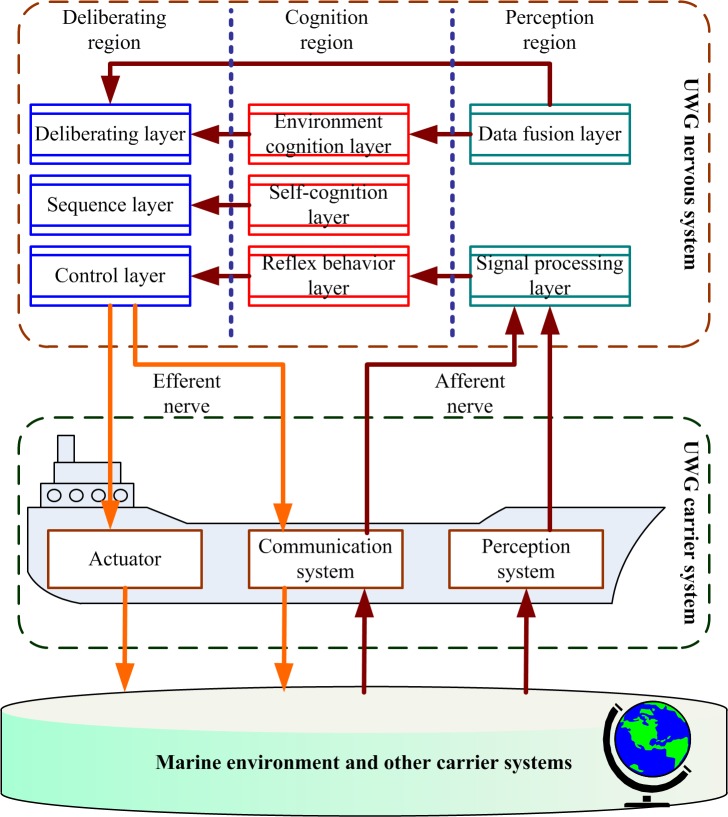
Neuropsychological structural model of UWG.

Based on the cerebrum basic function combination zone theory, the nervous system is generalized as three basic combination zone: deliberating region, cognition region, and perception region, and each region consists of three cortexes (Fig 3).

Detailed introduction of the nervous system of UWG:

Perception region is composed of signal processing layer, data fusion layer, and pattern recognition layer (ignored, UWG is not able to carry radar or visual environment monitoring devices limited by power capacity). Perception system or communication system creates nervous impulse, and transfer to the signal processing layer by afferent nerve. Sensors data is preprocessed, digital filtered, fault detected and diagnosed in the signal processing layer and then is transferred to the reflex behavior layer and the data fusion layer. The data fusion layer is responsible for data fusion of the results from signal processing layer and transfers to the environment cognition layer and deliberating layer.

Cognition region is composed of reflex behavior layer, self-cognition layer, and environment cognition layer. The environment cognition layer is responsible for maintaining marine environment model. The self-cognition layer is responsible for maintaining the self-cognition model of UWG. The above-mentioned two kinds of cognition information are provided for the sequence layer and deliberating layer. The reflex behavior layer is composed of pre-established reflex behavior databases which are contains mappings from various kinds of special conditions to basic survival principles. When malfunction or dangerous events happen, UWG must make quick response and send the information to the control layer and self-cognition layer, and takes different reflex behaviors (emergency steering, communication for help, etc.) based on basic survival principles. High reliability and security are necessary for an UWG, and the corresponding control strategies aimed at actuator faults, sensor faults, emergency events are mainly concerned. Considering the fault tolerance, the reflex behavior layer should guarantee the reliability of UWG as much as possible.

Deliberating region is composed of deliberating layer, sequence layer, and control layer. The deliberating layer is the core of UWG’s cognitive psychological activities and is composed of desires, convictions, capacities, commitment principles, commitments, intentions, and learning mental modules etc. Desires contain the task goals of UWG which can be obtained by communication; convictions contain information about marine environment, UWG itself and other members in UWG fleets; capacities are the executable actions of UWG. UWG’s cognitive psychological activities are mainly derived by targets but not only triggered by environment, which means that the UWG’s mind is active. According to the intentions from the deliberating layer, the sequence layer plans out the behavior sequence. Based on the behavior sequence from the sequence layer, the control layer creates communication information using communication system or directly controls the actuators.

**Fig 4:**
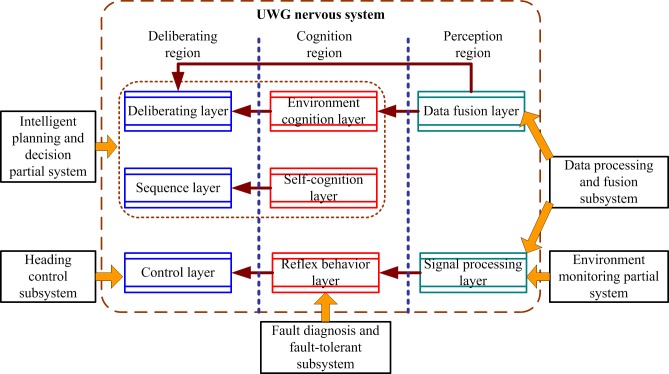
Inter system correspondences.

Obviously, the partial systems of the UWG intelligent control system is an organic integrity abstracted from the layers of the nervous system. For example, the data processing and fusion partial system corresponds to the signal processing and data fusion layer, and the heading control subsystem corresponds to the control layer. The detailed correspondences are shown in Fig 4.

The UWG’s motion control partial system is the core of UWG’s intelligent control system and will be mainly concerned.

### Hardware design of the motion control system

**Fig 5:**
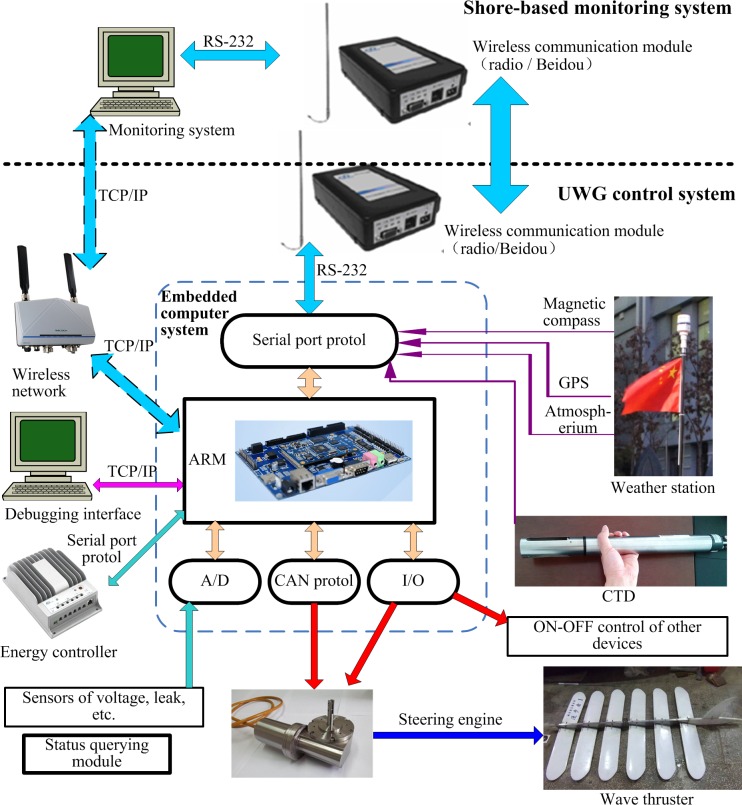
Hardware architecture of UWG’s motion control system.

UWG control system consists of embedded computer system (hardware) and embedded control system (software). The core of embedded computer system is a development board based on Atmel industrial processor (embedded arm computer system). The development board highly integrated ARM9 core of 400MHz and provide abundant peripheral interfaces: ADC, CAN, serial port, Ethernet, USB, GPIO, PWM, SDIO, voice interface, audio signal interface, video signal interface, TF card interface etc. Compared to other X86 embedded computer system like PC/104, industrial ARM computer guarantees the reliability and environmental suitability in long-time running and reduces the power consumption. Fig 5 shows the hardware architecture of UWG control system.

Considering the expandability and cost, we choose the open source multiple task operating system Linux in embedded ARM computer. Linux is stable, tailorable and convenient to develop and use, it is widely used in many fields like communication system, industrial control, aerospace, consumer electronics etc. Shore-based monitoring system is developed using Matlab GUI under Windows. By wireless communication system, the shore-based monitoring computer can monitor the working condition of UWG remotely at real time. Besides, network debugging and monitoring interface are reserved.

### Software design of the motion control system

**Fig 6:**
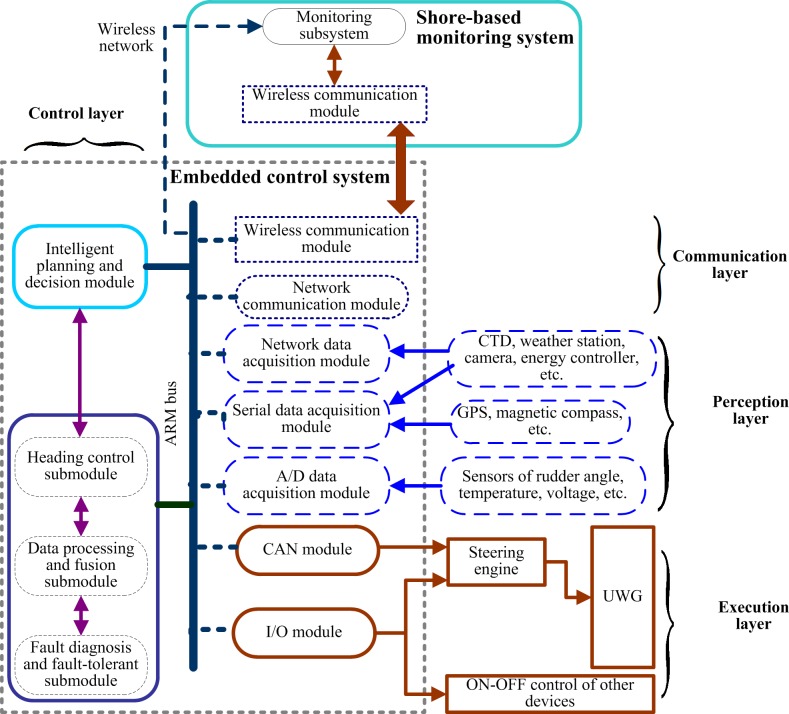
Software architecture of UWG’s motion control system.

The embedded control system of UWG consists of environment and motion perceptive module, control module, wireless data communication module, execution module, as shown in Fig 6.

Combining space-time decomposition architecture and behavioral response architecture, the layered UWG embedded control system is divided into four layers: control layer, communication layer, perception layer, execution layer. The main structure and function of each layer: (1) control layer: includes motion control sublayer and intelligent decision sublayer. Motion control sublayer consists of heading control submodule, data processing and fusion submodule, fault diagnosis and fault-tolerant control submodule. Motion control sublayer processes environmental and position information, and makes control commands according to target commands, and is the core of embedded control system. Intelligent decision sublayer is the “brain” of UWG. Intelligent decision sublayer cognizes the environment and itself, goal driven decides the behavior intention, and plans the action sequence, and finally makes target commands. Risk avoiding and special task modules are also integrated into the control layer and coordinated by intelligent decision sublayer. (2) communication layer: directs the communication of the whole system, including wireless serial port communication, TCP/IP network communication, ARM bus communication etc. (3) perception layer: namely, environment and motion perceptive module, including some data collecting modules like A/D card, serial card, network, and collects sensor data about environment, position and attitude. (4) execution layer, namely execution module, understands control commands to send to actuators and devices. It mainly consists of CAN module and I/O card.

## Navigation, Guidance and Control Methods

The speed of UWG is uncontrollable, so it is difficult to following the path directly. We choose the indirect path following method—according to the desired position of UWG and based on specific guidance algorithm and obtain the desired heading command, to realize the UWG path following. The motion control system controls the rudder system to track the desired heading and finally the path following is realized indirectly.

**Fig 7:**
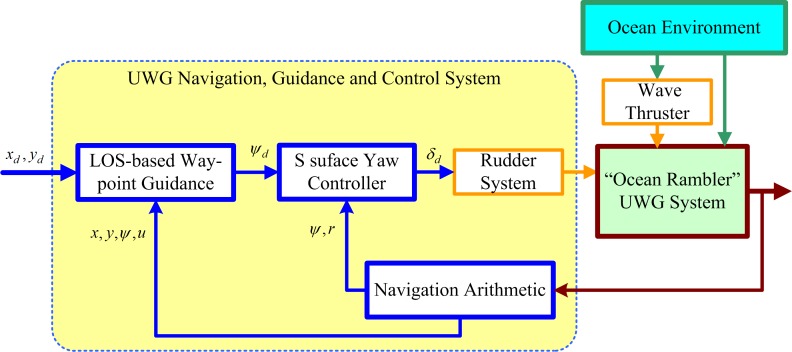
Schematic diagram of UWG navigation, guidance and control system.

The whole system operates as shown in Fig 7:

1. According to desired waypoint coordinates (*x*_*d*_,*y*_*d*_) and current location coordinates and motion states (heading angle, longitudinal velocity) of UWG (*x*,*y*,*ψ*,*u*), the desired heading *ψ*_*d*_ is calculated by LOS-based way-point guidance algorithm;
2. Desired heading *ψ*_*d*_ is transmitted to S surface yaw controller. The command rudder angle *δ*_*d*_ is calculated;
3. Rudder system takes action according to the command rudder angle;
4. UWG motions in real environment. There are complex forces act on UWG including environmental forces, wave thrust force and rudder force;
5. Current location and motion states of UWG (heading angle, longitudinal velocity, turning speed) (*x*,*y*,*ψ*,*u*,*r*) is measured by sensors. Motion parameters are calculated using a certain navigation arithmetic. Then the motion parameters are transmitted to the guidance part;
6. Return to step (1);

This work mainly focuses on guidance law and controller design.

### Self-adaptive guidance algorithm

Because UWG mainly sails in open sea, the expected route is usually straight line or way-point path, which is composed of some line segments. We consider a generic desired geometric path composed of a collection of way-points (…,***P***_*k*−1_,***P***_*k*_,***P***_*k*+1_,…).

**Fig 8:**
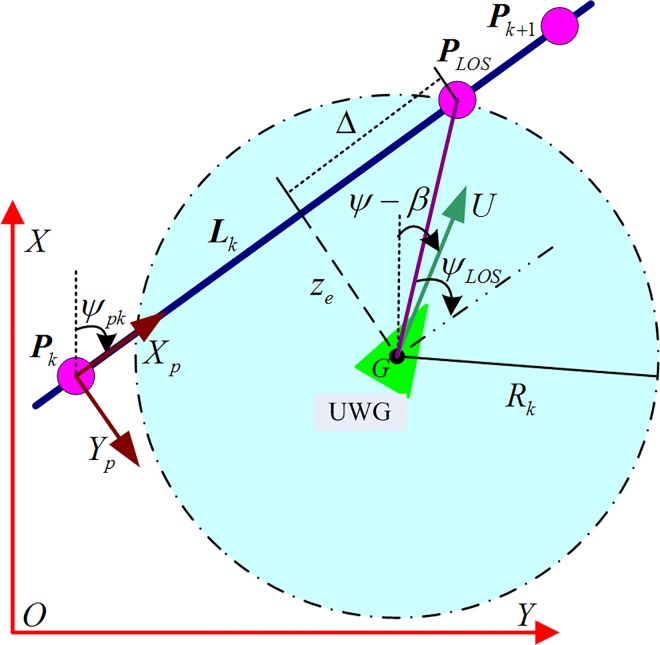
Illustration of the UWG straight-line path following.

As shown in Fig 8, consider a straight-line path $L_{k}\in \vec{P_{k}P_{k+1}}$ implicitly defined by two way-points through which it passes. Denote these way-points as ***P***_*k*_ = [*x*_*k*_,*y*_*k*_]^T^ ∈ ℝ^2^ and ***P***_*k*+1_ = [*x*_*k*+1_,*y*_*k*+1_]^T^ ∈ ℝ^2^, respectively. Simultaneity, consider a path-fixed reference frame with origin in ***P***_*k*_, whose x-axis has been rotated a positive angle *ψ*_*pk*_ = atan2(*y*_*k*+1_−*y*_*k*_,*x*_*k*+1_−*x*_*k*_)∈[−*π*,*π*] relative to the x-axis of the stationary reference frame [27,28], *ψ*_*pk*_ can be obtained from Eq 1; In Fig 8, *ψ*_*pk*_ is included angle between the straight path ***L***_*k*_ and coordinate axis X. ${\dot{\psi }}_{pk}=0$; *z*_*e*_ is the length between the barycenter of UWG(point M) and ***L***_*k*_, (cross-track error); *ψ*_*e*_ = *ψ*−*ψ*_*pk*_ refers to relative heading error; $U=\sqrt{u^{2}+\upsilon^{2}}$ expresses the resultant velocity of UWG; *β* is included angle between longitudinal velocity *u* and resultant velocity(sideslip angle).

$$\text{atan}2(\rho ,\sigma )=\{\begin{array}{l} \;\arctan(\frac{\rho }{\sigma })\;\sigma >0\\ \;\pi +\arctan(\frac{\rho }{\sigma })\;\rho \geq 0,\sigma <0\\ -\pi +\arctan(\frac{\rho }{\sigma })\;\rho <0,\sigma <0\\ \;\frac{\pi }{2}\;\rho >0,\sigma =0\\ \;-\frac{\pi }{2}\;\rho <0,\sigma =0 \end{array}$$ (1)

In fact when operating UWG, transverse velocity is much smaller than longitudinal velocity, thus it can be ignored, as a result *υ* = 0, *β* = 0. So the error dynamics of liner path can be expressed as follows:

$$\left\{ \begin{array}{l} {\dot{z}}_{e}=u\sin\psi_{e}^{}+\upsilon \cos\psi_{e}^{}\\ {\dot{\psi }}_{e}^{}=r \end{array}\right.$$ (2)

Line of sight guidance is widely used in the liner path tracking such as the ships, unmanned crafts and underwater robots [27–30]. LOS angle can be obtained from Eq 3:

$$\psi_{\text{LOS}}=\text{arctan}\left(-\frac{z_{e}}{\Delta }\right)$$ (3)

*ψ*_LOS_ ∈ [−*π*/2,*π*/2]. Δ expresses look ahead distance, and Δ > 0.

If a UWG heading follows exactly what is specified by the LOS algorithm Eq (3), one can establish the desired path following performance. Note, from Eq (2), that

$${\dot{z}}_{e}=u\sin\psi_{e}^{}+\upsilon \cos\psi_{e}^{}=U\sin\left(\psi_{e}+\arctan\frac{\upsilon }{u}\right)$$ (4)

If the heading of the UWG *ψ*_*e*_ is made to satisfy

$$\psi_{e}+\arctan\frac{\upsilon }{u}=\psi_{\text{LOS}}$$ (5)

Then we have

$$\begin{array}{l} {\dot{z}}_{e}=U\sin\psi_{\text{LOS}}\\ \;=U\sin\left[\text{arctan}\left(-\frac{z_{e}}{\Delta }\right)\right]\\ \;=-\frac{U}{\sqrt{z_{e}^{2}+\Delta^{2}}}z_{e} \end{array}$$ (6)

Define Lyapunov function as

$$V_{1}=\frac{1}{2}z_{e}^{2}$$ (7)

Differentiating *V*_1_ along the solution of Eq (2), and substituting Eq (6) into it yields

$${\dot{V}}_{1}=-\frac{U}{\sqrt{z_{e}^{2}+\Delta^{2}}}z_{e}^{2}\leq 0\;(\text{if},\;U>0)$$ (8)

Therefore, from Eq (8) it can be derived that the cross-track error *z*_*e*_ is globally asymptotically stable if the UWG’s absolute speed $U=\sqrt{u^{2}+\upsilon^{2}}$ is positive.

In 2008, Breivik et al. [30] shows that the disturbance of the marine environment (especially continuous currents) has a negative effect on the motion of the marine vehicles(especially under-actuated systems). In path following tasks, conventional LOS guidance methods will lead to steady state error inevitably. Based on LOS and PID methods, a PID-LOS guidance method is designed to compensate the adverse effects of the currents.

$${\bar{\psi }}_{\text{LOS}}=\text{arctan}\left(\text{-}k_{p}•z_{e}\text{-}k_{i}•\int_{0}^{t}z_{e}(\tau )d\tau \text{-}k_{d}•{\dot{z}}_{e}\right)$$ (9)

${\bar{\psi }}_{\text{LOS}}$ is correctional LOS angle and is the function of side deviation *z*_*e*_. ${\bar{\psi }}_{\text{LOS}}$ is obtained by real-time calculation using PID method. *k*_*p*_,*k*_*i*_,*k*_*d*_ are guidance parameters, and they are all positive.

Based on the research experience of the PID-LOS method, an self-adaptive PID-LOS guidance method is proposed named APID-LOS. Self-adaptive adjustment strategies of guidance parameters are as follows:

1. According to the path following error, the path following process is divided into three stages: pointing stage(*z*_*e*_ ≥ *n*_larger_*L*_ship_), closing stage (*n*_little_*L*_ship_ ≥ *z*_*e*_ > *n*_larger_*L*_ship_) and stable following stage (*z*_*e*_ < *n*_little_*L*_ship_). *n*_larger_,*n*_little_ are design parameters which are determined according to the size and the motor performance of the float.
2. Pointing stage—the proportional is dominant, while ignores the role of differential and integral. In this stage, ${\bar{\psi }}_{\text{LOS}}=\psi_{\text{LOS}}$ is in P type. The proportion parameter increases with the increase of error to speed up the response of the system. In this paper we choose the parameters $k_{p}=20\;\tanh\frac{\left|z_{e}\right|}{100}+{\bar{k}}_{p}$, *k*_*i*_ = *k*_*d*_ = 0.
3. Closing stage—proportional and differential are dominant while ignores the integral. The purpose is to restrain overshoots. In this stage, ${\bar{\psi }}_{\text{LOS}}$ is in PD type. In this paper we choose $k_{p}=20\;\tanh\frac{\left|z_{e}\right|}{100}+{\bar{k}}_{p},k_{i}=0,k_{d}={\bar{k}}_{d}$.
4. Stable following stage—proportional, differential and integral work together to select the appropriate integral coefficient to eliminate steady-state error. In this stage the ${\bar{\psi }}_{\text{LOS}}$ is in PID type. In this paper we choose $k_{p}={\bar{k}}_{p},k_{i}={\bar{k}}_{i},k_{d}={\bar{k}}_{d}$.
5. Taking actual control into account, the integration is obtained by numerical methods. In order to avoid integral saturation, the tracking error $Z_{\mathrm{int}}=\int_{0}^{t}z_{e}(\tau )d\tau$ is obtained according to Eq (10).

$$Z_{\mathrm{int}}=\left\{ \begin{array}{l} \sum_{i=1}^{n}z_{e}(i)\;(Z_{\mathrm{int}}\leq Z_{\max})\\ \sum_{i=1}^{n}z_{e}(i)\;(Z_{\mathrm{int}}>Z_{\max},z_{e}•{\dot{z}}_{e}<0)\\ Z_{\mathrm{int}}\;(Z_{\mathrm{int}}>Z_{\max},z_{e}•{\dot{z}}_{e}\geq 0) \end{array}\right.$$ (10)

*Z*_max_ = *n*_max_*L*_ship_ is upper limit of the integration which is determined by the size of the float.

### Improved S-surface controller

Table 1 shows the performance comparison between the “Ocean Rambler” UWG and some USVs and UUVs. Data of the “Ocean Rambler” UWG in Table 1 are obtained by tank trials. The “XL” USV, “WeiLong” UUV and “HaiLing” UUV in Table 1 are designed by the Science and Technology on Underwater Vehicle Laboratory in Harbin Engineering University, and the data is provided by the designers. Data of “USV14” USV is obtained from [31].

**Table 1:** Performance comparison between the “Ocean Rambler” UWG and some USVs and UUVs.

	“USV14” USV	“XL” USV	“WeiLong” UUV	“HaiLing” UUV	“Ocean Rambler” UWG
Length(m)	4.3	6.2	2.5	4.3	3(float)
Propulsion type	electric thruster	Pump-jet	electric thruster	electric thruster	wave thruster
Turning diameter(m)	12	≈15	≈18	≈14	≈30
Highest speed(m/s)	2.8	16	2.5	2.5	0.3–0.5(SS1) 0.8–1.0(SS3)
Turning delay(s)	/	≈1	≈1.2	≈1	≈5

From Table 1 and practical experiences, we know that compared with traditional unmanned vehicles, UWG is with larger turning diameter, lower highest speed and longer turning delay. The low speed of UWG causes bad steerage and weak heading control ability.

In a word, the UWG system is weak maneuvering, large time-lag and large disturbance, which is difficult to establish accurate mathematical model even heading response equation. And the designed controller must satisfy the characteristics of UWG. Because of the special structure, it is difficult for UWG to take tank trials or sea trials. Considering about shortening the debugging time and saving experimental expense, a controller that is simple, efficient and easily to adjust parameters is needed. Besides, the designed controller must be robust to complex environmental forces.

Intelligent control combines control theory and artificial intelligence technology (AIT) together flexibly, and can be applied on complex and uncertain controlled members. Optional intelligent control methods include neural network, fuzzy logic control, expert control, etc. In this paper, we choose S-surface controller [32]. S-surface controller combines fuzzy control theory and simple PID control structure together and is widely used in the control of underwater vehicle, USV etc.[32–34]

Description of S-surface controller is as follows: take the numerical values from the principle diagonal of fuzzy controller control rule table (Table 2) and then link to a fold line. The fold line can be fitted as a smooth curve (like tanh function, Sigmoid function etc.). Actually the smooth curve can be seen as a fold line with innumerable pieces while the length of each piece tends to zero. Obviously, the fold line surface that corresponds to the whole fuzzy control law library can be fitted by a smooth curved surface.

**Table 2:** Control rule table.

4	3	2	1	0
3	2	1	0	-1
2	1	0	-2	-2
1	0	-1	-2	-3
0	-1	-2	-3	-4

The Sigmoid curve function can be expressed as:

$$u_{c}=2.0/(1.0+e^{-kx})\;\text{-}\;1.0$$ (11)

Then, the Sigmoid curved surface function is:

$$z=2.0/(1.0+e^{-k_{1}x-k_{2}y})-1.0$$ (12)

**Fig 9:**
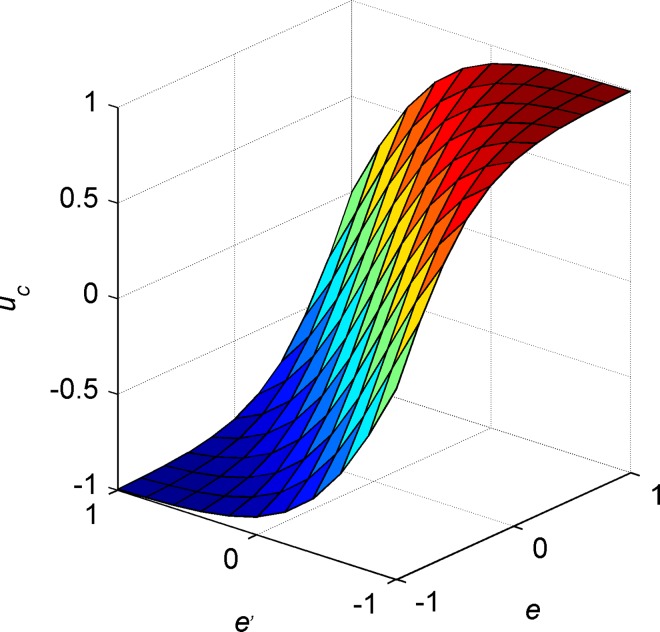
Sigmoid curved surface.

We choose the control model of the S-surface controller as:

$$u_{c}=2.0/(1.0+e^{-k_{1}e-k_{2}\dot{e}})-1.0$$ (13)

where *e* and $\dot{e}$ are control inputs (normalized error and error rate respectively); *u*_*c*_ is the normalized control force output; *k*_1_, *k*_2_ are the control parameters corresponding to error and error rate respectively, and can influence the rate of change of corresponding control inputs. The three-dimensional smooth curved surface in Fig 9 shows the relationship between error, error rate and control force. Compared with PID controller, the structure of S-surface controller is similar to the structure of PD controller, but PD controller is linear and S-surface controller is nonlinear. Commonly, using a nonlinear function to fit nonlinear system is more accurate than using a linear function [33].

By adjusting *k*_*i*1_ and *k*_*i*2_, the share of error and error rate in control output can be changed. In other words, the control characteristics like overshoot and rate of convergence can be changed to meet the control demands by adjusting *k*_*i*1_ and *k*_*i*2_. The usual control parameters adjusting laws: if the overshoot is large, decrease *k*_*i*1_ and increase *k*_*i*2_; otherwise, if the system converges slowly, increase *k*_*i*1_ and decrease *k*_*i*2_.

We propose a dynamic parameter adjustment method. The basic idea is that under large error, a large proportion parameter and a little differential parameter are chosen to accelerate the system response; under little error, a little proportion parameter and a large differential parameter are chosen to improve the dynamic control performance. As to a speed or heading controller, a simple adjustment law is as follows as an example.

$$\begin{array}{l} k_{r1}=\left\{ \begin{array}{l} 2{\bar{k}}_{r1}\;(\psi_{e}\geq 0.5{\bar{\psi }}_{\max})\\ 0.5{\bar{k}}_{r1}\;(\psi_{e}<0.5{\bar{\psi }}_{\max}) \end{array}\right.\\ k_{r2}=\left\{ \begin{array}{l} 0.5{\bar{k}}_{r2}\;(\psi_{e}\geq 0.5{\bar{\psi }}_{\max})\\ 2{\bar{k}}_{r2}\;(\psi_{e}<0.5{\bar{\psi }}_{\max}) \end{array}\right. \end{array}$$ (14)

*ψ*_*e*_ is heading control error; ${\bar{\psi }}_{\max}$ is the normalized scale in heading control; ${\bar{k}}_{r1},{\bar{k}}_{r2}$ are nominal proportion and integration parameters; *k*_*r*1_,*k*_*r*2_ are adjusted proportion and integration parameters.

## Trial Results and Analysis

### Simulation results and analysis

It is difficult to establish the mathematical model for UWG, and the commonly accepted mathematical model had not been formed up to now. In this paper, we take a mini USV as an example to carry out simulation experiments to primarily verify the effectiveness of the above-mentioned control methods. The length of the USV *L*_ship_ = 3m, and its main parameters are as follows [35]:

$$\begin{array}{l} m_{11}=200\text{kg},\;m_{22}=250\text{kg},m_{33}=80\text{kg}\cdot {\text{m}}^{2},\\ d_{11}=70\text{kg}/\text{s},\;d_{22}=100\text{kg}/\text{s},\;d_{33}=50\text{kg}\cdot {\text{m}}^{2}/\text{s}. \end{array}$$

**Fig 10:**
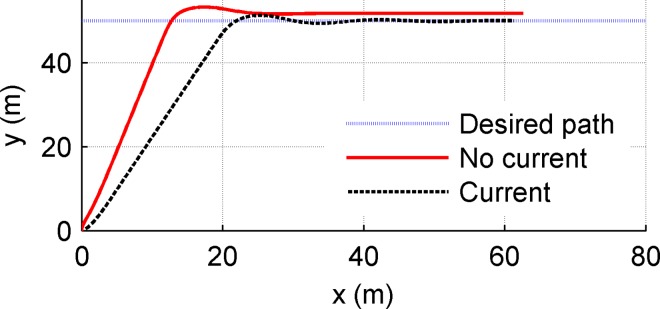
Simulation results using LOS guidance method.

**Fig 11:**
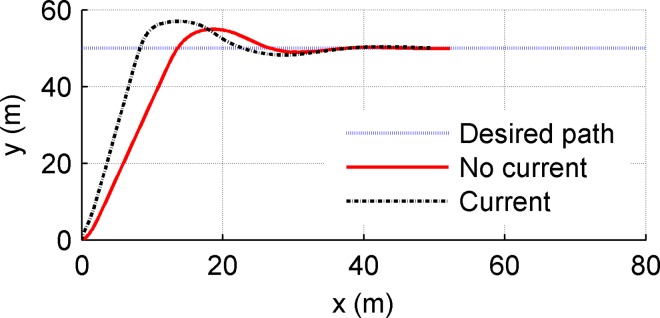
Simulation results using APID-LOS guidance method.

The parameters *m*_11_,*m*_22_,*m*_33_ are the vehicle inertial including additional masses, and *d*_11_,*d*_22_,*d*_33_ denote the hydrodynamic damping parameters. Set the desired path as a straight line *x*_*d*_ = 0.5*t*,*y*_*d*_ = 50; set two conditions: (1)no current (*V*_*current*_ = 0), (2)Considering an extreme condition: constant current perpendicular to the setting path(*V*_*current*_ = 0.3m/s,*ψ*_*current*_ = 90°); desired cruising speed is set as 0.5m/s. The initial conditions are set as (*x*(0),*y*(0),*ψ*(0),*u*(0),*υ*(0),*r*(0)) = (0,0,0,0,0,0); main control parameters are chosen as ${\bar{k}}_{p}=2.5,{\bar{k}}_{i}=2,{\bar{k}}_{d}=0.5$, $n_{\text{larger}}=20,n_{\text{little}}=5,n_{\max}=100,{\bar{k}}_{u1}=6.5,{\bar{k}}_{u2}=5,{\bar{k}}_{r1}=9,{\bar{k}}_{r2}=1$. The contrast simulation experiments are carried out using the LOS and APID-LOS guidance methods respectively. The results are shown in Figs 10 and 11.

Fig 10 shows the path following simulation results using LOS guidance method. There are three lines in Fig 10. The three lines represent the desired path and actual path(no current and with current) respectively as the legend shows. Fig 11 is similar to Fig 10 while using APID-LOS guidance method. From Figs 10 and 11, in no current condition, the USV can track the desired path guided by LOS and the overshoot is 1.2m; in contrast, the overshoot using APID-LOS is about 4.9m which is larger. Both the two methods can converge to the desired path with zero state error, and the dynamic performances are good. In the second condition, the velocity and direction of the currents are with typical significance: Currents perpendicular to the desired path is the worst for USV’s tracking operation; 0.3m/s is the common current velocity of surface currents close to China’s coast. When the speed of the current reaches 60% of the cruising speed of the vehicle, it is challenging for a mini USV. Under the influence of the current, the USV cannot converge to the set path using LOS method, and the steady-state error is 2m; using APID-LOS method, the USV finally converge to the desired path with zero deviation and completes the path tracking task. Compared with the first condition, the stabilization time is equivalent, but the overshoot reaches 6.9m which is larger.

### Autonomous path following trial results and analysis

In 2015, “Ocean Rambler” UWG completed sea trials in Weihai coastal waters in China. The sea trial area is managed by Xixiakou Tourism Company, and we have obtained the permission from the manager. Energy resources of UWG are completely clean and there is no pollution to the marine environment. There is no propeller and other dangerous parts to marine organism, so the Unmanned Wave Glider is safe to marine organism.

**Fig 12:**
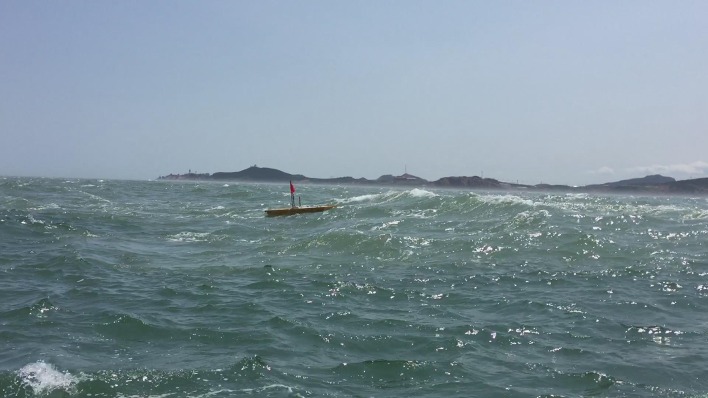
Sea trials of “Ocean Rambler” UWG.

Main trial subjects include: remote controlled sailing, motion control, autonomous path following, autonomous environment monitoring, etc. The raw data can be obtained in S1 Table. Fig 12 shows the “Ocean Rambler” UWG sailing in sea trials. Part of the trial results and analysis are shown below:

The “Ocean Rambler” UWG is with displacement of 130kg, length of 3m and width of 0.7m. The above- mentioned intelligent control system is deployed on the UWG. The desired path is composed of four line segments (contains five key way-points shown in Table 3).

**Table 3:** Longitude and latitude of desired way-points.

sequence	longitude(°)	latitude(°)
1	122.658	37.4539
2	122.6902	37.441
3	122.7248	37.4253
4	122.7326	37.4031
5	122.7365	37.3914

**Fig 13:**
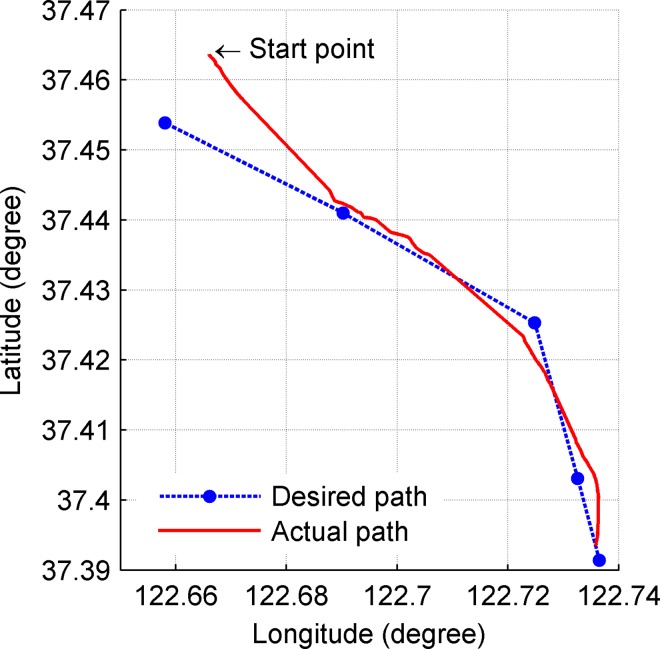
Actual path and desired path.

**Fig 14:**
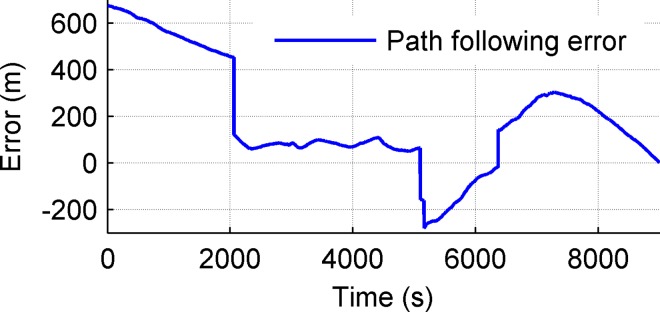
Path following error.

Other than traditional unmanned marine vehicles, the speed of UWG is decided by marine environment, which means that it does not involve speed control problem. The starting point of the desired path is longitude 122.666°and latitude 37.464°; Main control parameters are chosen as ${\bar{k}}_{p}=2.5$, ${\bar{k}}_{i}=2$, ${\bar{k}}_{d}=0.5$, *n*_larger_ = 200,*n*_little_ = 50, $n_{\max}=200,{\bar{k}}_{r1}=9$, ${\bar{k}}_{r2}=1$. Sea trials were carried out in SS3 sea condition and the wind reached Force 2–3. Autonomous path following trial results are shown in Figs 13 and 14.

Sea trials lasted for 4.2 hours. The length of the desired path was 13km and the average velocity of UWG was 0.85m/s. There are two lines in Fig 13. The blue dotted line represents the desired path. The read solid line represents the actual path. Fig 14 shows the path following error changing over time. The geometrical significance is shown in Fig 8 which is vertical distance to the desired path. The desired path of UWG is composed of four line segments. The desired path changes three times at 2110s, 5160s and 7179s which causes mutations of the path following error. From Figs 13 and 14, the initial tracking error was 680m and the UWG finally completed path following tasks with steady-state error less than 300m. Trial results show that the UWG has relatively strong path following ability in Marine environment which lays the foundation for autonomous marine environment monitoring tasks.

It should be noted that compared with traditional unmanned vehicles, UWG is weak maneuvering, low speed (about 0.5–2kn) and the speed is uncontrollable. It causes bad steerage and poor ability to defend environmental disturbances especially currents. The path following error of traditional vehicles or USV is meter-scale or decametric scale but with weak endurance, so it is proper to make short-range and detailed environment monitoring for traditional vehicles or USV. The path following error of UWG is hundred-meter scale but with virtually unlimited endurance, and tracking error is acceptable for large-scale marine environment monitoring tasks where detection nodes are hundreds kilometers apart, so it is proper to make large-scale environment monitoring applications.

### Autonomous environment monitoring trial results and analysis

**Fig 15:**
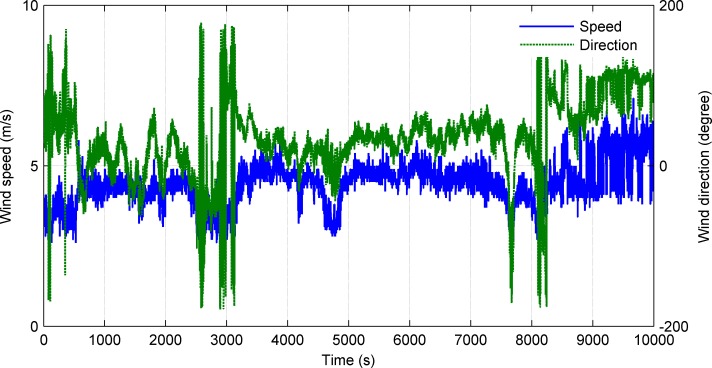
Wind speed and wind direction.

**Fig 16:**
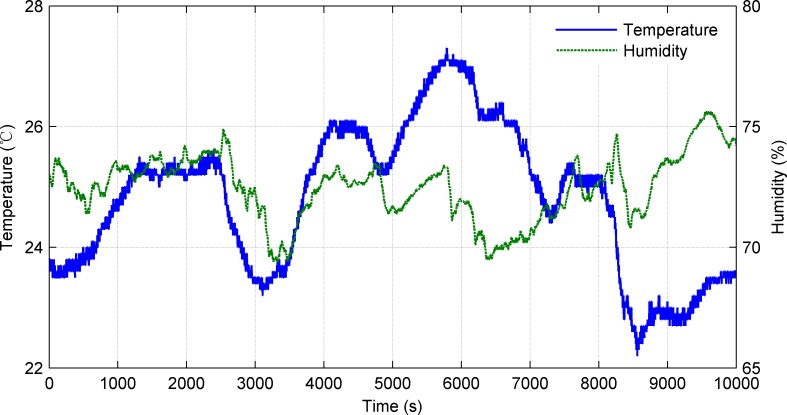
Air temperature and relative humidity.

**Fig 17:**
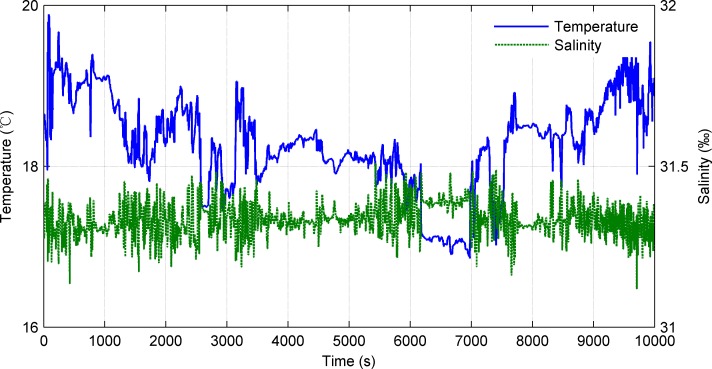
Seawater temperature and salinity.

The mission payloads on the UWG are an integrated weather station and a CTD. In the sea trials, we chose automatic mode of the weather station. The weather station was fastened to the mast on the float and 1.2m off the sea surface. The CTD was deployed on the glider 5.8m below the sea surface. Parts of the collected meteorological data are shown in Figs 15–17.

Figs 15 and 16 show the meteorological data along the traveled path obtained by weather station. Fig 15 shows the wind speed and wind direction data. The average wind speed is 4.1m/s and the wind direction fluctuates between 15–80°(mainly northeast wind). Fig 16 shows the air temperature and relative humidity. The average air temperature is 24.8°C and the average relative humidity is 74.5%. Besides, data of sea surface temperature, salinity, conductivity were obtained in trials. Fig 17 shows the sea surface temperature and salinity data. The average sea surface temperature is 18.3°C and the average salinity is 31.3‰. The UWG can percept the meteorological differences in the experimental sea area (7km×7km) which proves the good environment monitoring of the UWG. Space and electric interfaces are reserved on the UWG. In the future, according to the application requirements the UWG can carry multi kinds of sensors like ADCP, wave gauge, water quality sensor, etc.

The sea trial results show that the UWG carrier, intelligent control system, actuator, electric devices, sensors, and monitoring payloads work steadily and effectively which verifies the feasibility and conscious control ability of UWG. It set the technical and platform foundation for further research.

## Conclusions

The intelligent control system of “Ocean Rambler” UWG was completed and sea trials including autonomous path following and environment monitoring were carried out. Trial results prove that the designed intelligent control system can achieve autonomous path following control and environment monitoring tasks, and the path following ability is pretty good. (2) The designed guidance strategies and control methods don’t rely on the mathematical model of UWG. They have strongly adaptability for uncertainties and can be easily extended to applications. (3) the intelligent control system work steadily during the experiments. The trial results verify the validity and feasibility of the intelligent control system in aspects of architecture, guidance algorithm, control algorithm, data processing, operational software and hardware.

Only tank trials in low sea condition were completed. The future work will focus on: combining the intelligent control theory and current guidance and control methods together to improve the control performance and adaptive ability in rough marine environment; the measurement of wind speed and wind direction is disturbed severely by large shock of UWG’s speed and course, and the influencing mechanism and compensation method need to be discussed; it is necessary to carry out large-scale sea trials including autonomous path following and autonomous environment monitoring in high sea states.

## Supporting Information

S1 Table (XLS)
