# Expression of Concern: The Intelligent Control System and Experiments for an Unmanned Wave Glider

**DOI:** 10.1371/journal.pone.0268056

**Published:** 2022-04-28

**Authors:** The PLOS ONE Editors

This article [1], including the title and parts of the main text, uses the term “wave glider” generically to refer to unmanned surface vessels. Liquid Robotics, Inc. claims trademark rights to the term “wave glider” and as such the term is not a generic description for this class of vehicle.

During editorial follow up about the above, the corresponding author indicated that the software for the numerical simulation of the guidance algorithm is no longer available. The corresponding author stated that this article concentrates on the control system design and the sea trial testing of the unmanned surface vessel. They noted that the main purpose is to verify the reliability of the control system and the performance for path following and environmental monitoring by sea trials.

The PLOS Editors consider that the current unavailability of the software code impacts this article’s adherence to *PLOS ONE*’s publication criteria regarding reproducibility. In light of this, the *PLOS ONE* Editors issue this Expression of Concern.
